# *Glycyrrhiza uralensis* Fisch. Root-associated microbiota: the multifaceted hubs associated with environmental factors, growth status and accumulation of secondary metabolites

**DOI:** 10.1186/s40793-022-00418-0

**Published:** 2022-05-07

**Authors:** Chaoyun Chen, Chaofang Zhong, Xi Gao, Chongyang Tan, Hong Bai, Kang Ning

**Affiliations:** Key Laboratory of Molecular Biophysics of the Ministry of Education, Hubei Key Laboratory of Bioinformatics and Molecular-Imaging, Center of AI Biology, Department of Bioinformatics and Systems Biology, College of Life Science and Technology, Huazhong University of Science and Technology, Wuhan, 430074 Hubei China

**Keywords:** *Glycyrrhiza uralensis* Fisch., Root-associated microbiota, Plant growth promoting rhizobacteria, Secondary metabolites, Multi-omics

## Abstract

*Glycyrrhiza uralensis* Fisch. is an important, perennial medicinal plant whose root microbiome is considered to play an important role in promoting accumulation of effective medicinal ingredients (liquiritin and glycrrhizic acid). Here, we report a comprehensive analysis of the microbial community structural composition and metabolite-plant-microbes association of *G. uralensis* Fisch. We collected both soil and rhizosphere samples of *G. uralensis* from different environmental conditions (cultivated and wild) and growth years (grown for one year and three years). Our data revealed higher species diversity in the wild group than in the cultivated group. The core rhizosphere microbiome of *G. uralensis* comprised 78 genera, including *Bacillus*, *Pseudomonas*, *Rhizobium*, some of which were potential plant beneficial microbes. Our results suggest that the growth of *G. uralensis* has a correlation with the root-associated microbiota assemblage. Integrated analysis among rhizosphere microbial taxa, plant gene expressions, and liquiritin and glycrrhizic acid accumulation showed that the liquiritin and glycrrhizic acid accumulation exhibited associations with the rhizosphere microbial composition at the genus level. The results provide valuable information to guide cultivation of *G. uralensis,* and potentially to harness the power of the root-associated microbiota to improve medicinal plant production.

**Supplementary Information:**

The online version contains supplementary material available at 10.1186/s40793-022-00418-0.

## Background

Plant root-associated microbial communities, including endosphere, rhizoplane, rhizosphere and soil [1], benefit plants by preventing pathogenic infection and assisting in the acquisition of nutrition from the soil [2–4]. Understanding the taxonomic, genomic and functional components of root-associated microbial communities is crucial for their manipulation towards sustainable crop [5, 6]. Progress has been made toward the characterization of root-associated microbiota in certain crop plants by exploring the microbial community structure, core microbiome, and plant-microbiome interactions [7–9]. Meanwhile, there is little knowledge about how different assemblage pattern of root-associated microbiota can affect medicinal plant metabolome[10].

The recruitment of root-associated microbial community is largely driven by plant, and these microbes in turn have asserted great influences on the plant. Exudations from plants can be used to assemble root-associated microbial communities from the surrounding soil [11]. Among these exudations, sugars, amino acid, organic acids, fatty acids and secondary metabolites [12–14] like triterpenes [15] are of vital importance to the interactions between microbes and hosts. For example, it has been reported that the terpenoids secret by *Arabidopsis* were one of the carbon sources to the root-associated microorganisms [15]. Another study reported that root bacteria were found to consume aromatic organic acids secreted by A*vena* (nicotinic, shikimic, salicylic, cinnamic and indole-3-acetic) [16]. The complex plant–microbe interactions also have an impact on plant growth and yield[17]. For example, the reconstruction of the root microbial community promotes *Arabidopsis* survival [18]. Another work has reported that flavonoids secret by *Medicago truncatula* and then sensed by rhizobia would in turn produce Nod factors [19]. However, several questions remain elusive about the complex plant–microbe interactions, such as to what extent the plant itself assembles a microbial community from the surrounding soil, and how much influence of these microbes asserted on the plant growth and accumulation of secondary metabolites, especially the accumulation of active compounds of medicinal plant.

As an important medicinal plant, *Glycyrrhiza uralensis* Fisch. (the root and rhizome serving as the main medicinal materials) has been widely used to treat hepatitis, bronchitis, as well as malaria [20]. The main pharmacological components in the root of *G. uralensis* are glycyrrhizic acid and liquiritin [21]. The contents of glycyrrhizic acid and liquiritin vary greatly between different cultivation regions, and generally, the contents of glycyrrhizic acid and liquiritin of cultivated *G. uralensis* are lower than that of wild *G. uralensis* in China [22]. The factors that influence the growth of *G. uralensis* include nutrients, climate and even the root microbiome[23, 24]. How various biotic and abiotic factors influence the *G. uralensis* root-associated microbial communities, and how these microbes in turn affect the plant growth and accumulation of secondary metabolites for *G. uralensis*, are of great importance while remain poorly understood.

In this study, to decipher the association of the host metabolites with microbiota, we collected fresh *G. uralensis* root, rhizosphere and soil from *G. uralensis* that grew under cultivation and wild conditions, from Ningxia province of China. And we have generated metabolic, transcriptomic and microbial profiles for these samples. Multiple microbial diversity analysis of *G. uralensis* root-associated microbiota, as well as *G. uralensis* root multi-omics study including transcriptome and metabolite, were carried out to investigate the following questions: Firstly, how *G. uralensis* assembles a root-associated microbial community. Secondly, whether there is potential correlation between the root-associated microbial community structure and *G. uralensis* growth under different environments. Thirdly, how *G. uralensis* root-associated microbes associate with the accumulation of the glycyrrhizic acid and liquiritin.

## Materials and methods

### Sample collection

The samples were collected from Ningxia, China. The cultivated *Glycyrrhiza uralensis* Fisch grown for one year (C1) and three years (C3) were collected from village TianJiZhang (107.267819^o^E, 37.814875^o^N, of Yanchi County, Wuzhong City, Ningxia province). Wild *G. uralensis* were collected from village ShiJiQuan (106.861246^o^E, 37.98919^o^N, of Yanchi County, Wuzhong City, Ningxia province). The sampling methods vary according to the purpose of research. We dug out the *G. uralensis* and shook off the soil adhering to the *G. uralensis* root. The root was cut into about 10 cm fragments with scissors, that washed with 75% alcohol and dried naturally. The cut root samples were divided into three sections randomly (Additional file 1: Dataset 1, Table S1). One part of the three sections were collected in the germ-free centrifuge tubes immediately, which was defined as rhizosphere microbial sample in this study (“C1R” and “C3R” for the cultivated *G. uralensis* that were grown for one year and three years rhizosphere microbial samples, separately, and transport to the lab on dry ice. “WR” for the wild type *G. uralensis* rhizosphere microbial samples). The second part of the cut *G. uralensis* root was pre-freeze in the liquid nitrogen immediately for RNA-seq. While the left part was collected with a ziplock bag and dried naturally at room temperature in the following experiment for glycyrrhizic acid and liquiritin content determination. Besides that, the soil surrounding the root that shaken off from the *G. uralensis* was collected with germ-free centrifuge tubes for microbial research either, defined as *G. uralensis* soil microbial sample here (“C1S” and “C3S” for the cultivated *G. uralensis* that were grown for one year and three years soil microbial samples, separately. “WS” for the wild type *G. uralensis* soil microbial samples, Additional file 1: Figure S1). For every group of samples, about 25 replicates were collected, for example, 21 rhizosphere samples and 23 soil samples surrounding the *G. uralensis* root was collected for root-associated microbial profiling, 20 and 25 fresh *G. uralensis* root was collected for transcriptomic study and metabolite measurements, separately, for the cultivated *G. uralensis* that were grown for one year (Additional file 1: Table S1). All the microbial samples and the transcriptomic study samples were transported to the lab with dry ice.

### Microbial data collection and analysis

#### DNA extraction, 16S rRNA gene sequencing

As the first step to obtain the microbial raw data after sampling, the whole genome DNA was extracted using HiPure Soil DNA Kit B (Magen, China) step by step as the operation guide. The pre-processing methods for the soil microbial sample (soil surrounding the root that was shaken off from the *G. uralensis*) and the rhizosphere microbial sample were different, since the sample situation was totally different. For genome DNA extraction, 0.5 g of the soil samples was used. All the rhizosphere samples were firstly soaked with _dd_H_2_O for 4 h at room temperature followed by shaking the tubes with vortex (Vortex Genie2, USA) in the full speed. Thirdly, centrifuge the sample suspension at 5000 rpm for 10 min. And after that, the sediment was collected for DNA extraction as the rhizosphere microbial sample.

Acquiring the whole genome DNA suspension, the Qubit® dsDNA HS Assay Kit was used for DNA concentration measuring, monitored by Qubit3.0 Fluorometer. To generate the amplicons of the V3-V4 hypervariable regions of prokaryotic 16S rDNA, 20 ng genome DNA as template was used. The forward primers containing the sequence “CCTACGGRRBGCASCAGKVRVGAAT” and reverse primers containing the sequence “GGACTACNVGGGTWTCTAATCC” were used to get the V3-V4 amplicons. The 25 μL PCR reactions mixture contained 0.8 ng/μL of template DNA, 2.5 μL of TransStart Buffer, 2 μL of dNTPs, and 1 μL of each primer. Then, amplicons concentration was quantified by Qubit3.0 Fluorometer. Quantified to 10 nM according to manufacturer’s instructions (Illumina, San Diego, CA, USA), the quantified amplicons were multiplexed and loaded on an Illumina MiSeq PE 300 instrument. Image analysis and base calling were conducted by the MiSeq Control Software (MCS) embedded in the MiSeq instrument.

#### Microbial data Quality Control, OTU clustering, and taxonomy assignment

Mothur (version v.1.39.5) [25] was used for quality control and QIIME (V1.9.1) [26] was used for taxonomical analysis to obtain high-quality microbial data. The procedure was the same as [27, 28] in general: firstly, paired-end reads were spliced with ‘make.contigs’ command in the mothur with default settings. Secondly, removed all reads containing ambiguous base calls (N) and longer than 500 bp or shorter than 300 bp. After that, to identify putative chimeras with the SILVA database [29] as reference, ‘chimera.uchime’ command was used, followed by the removal of putative chimeras with ‘remove.seqs’ command. Besides, aligned by PyNAST [30], the high-quality sequences were clustered into unique representative sequences by UCLUST in QIIME. For operational taxonomic unit (OTU) classification (97% nucleotide identity), the Greengenes database (version 13_8) [31] was used as the reference database, and the minimum reads per OTU threshold was set as 2 for removing the singletons from the data.

#### Microbial diversity assessment and core microbiota profiling

Executed by the QIIME [26] pipeline, the microbial alpha-diversity and beta-diversity analyses were included in this study. For microbial community alpha-diversity profiling, rarefaction curves were drawn based on the richness metrics and evenness metrics. For beta-diversity analysis, Euclidean Distance (Supplementary Information), Jaccard Distance matrix were used to measure community similarity between samples. And, the statistics methods included student-test, Wilcoxon test and permutational multivariate analysis of variance (PERMANOVA, R package “Vegan” [32]). Microbial community clustering at different taxonomy levels was arrayed by principal coordinate analysis (PCoA) and visualized by package “ggplot” in R. Predictive functional profiling of campus microbial communities was generated by PICRUSt 1.1.0 from 16S rRNA marker genes. Meanwhile, more details about materials and methods for statistical analysis in taxonomical and functional prediction were provided in Supplementary Information (Statistics methods). For general scale sample difference analysis, the R package “cluster” was applied to the Jensen-Shannon Divergence (JSD) distance matrix. And the input data of the JSD distance matrix calculation was relative abundance (RA) table at genus level.

The microbe with a relative abundance greater than zero in no less than 50% of samples of the study group was defined as core microbe of that group. For the core rhizosphere microbiota, it was defined as intersection of core microbes of C1R, C3R, and WR. And co-occurrence plant growth promoting rhizobacteria (PGPR) in this study, either genus or species, was defined as the bacterium with RA greater than zero in no less than 50% of samples in that group.

### ﻿Multi-omics interaction analysis

The consistent analysis of the sample similarity of microbial and transcriptomics was performed with Procrustes Analysis. And the transcriptomics data collection and analysis methods were described in the Supplementary Information (Transcriptomic data collection and analysis). The input data for Procrustes Analysis included microbial RA table at genus level, fragments perkilobase million table (FPKM), and the metabolism absolute content table of glycyrrhizic acid and liquiritin. The Procrustes Analysis was performed in R with package “Vegan” [32] by function “procrustes” and “protest”, and the method “Euclidean” was applied to the sample distance calculation. In the further steps to study the statistical correlation of the multi-omics, Variance Partitioning Analysis was used. The input data for Variance Partitioning Analysis was the microbial community composition data at genus level and species level, and the accumulation of glycyrrhizic acid as well as liquiritin. And Variance Partitioning Analysis was performed in R with package “Vegan” by function “varpart”. To confirm the corresponding correlations between growth year and cultivation condition and root-associated microbiota assemblage, data on root-associated microbiota RA table at species level and the group information including plant growth and cultivation conditions were applied to build a Random Forest Binary Decision Tree called soil predictor. Area under the curve (AUC) of receiver operating characteristic curve (ROC) was used to evaluate the accuracy of this soil predictor. And, 80% of the samples were randomly chosen as a training dataset and the rest 20% of samples were used as the validation dataset.

## Results

### Characterization of the *G. uralensis* root-associated microbial communities

**Fig. 1:**
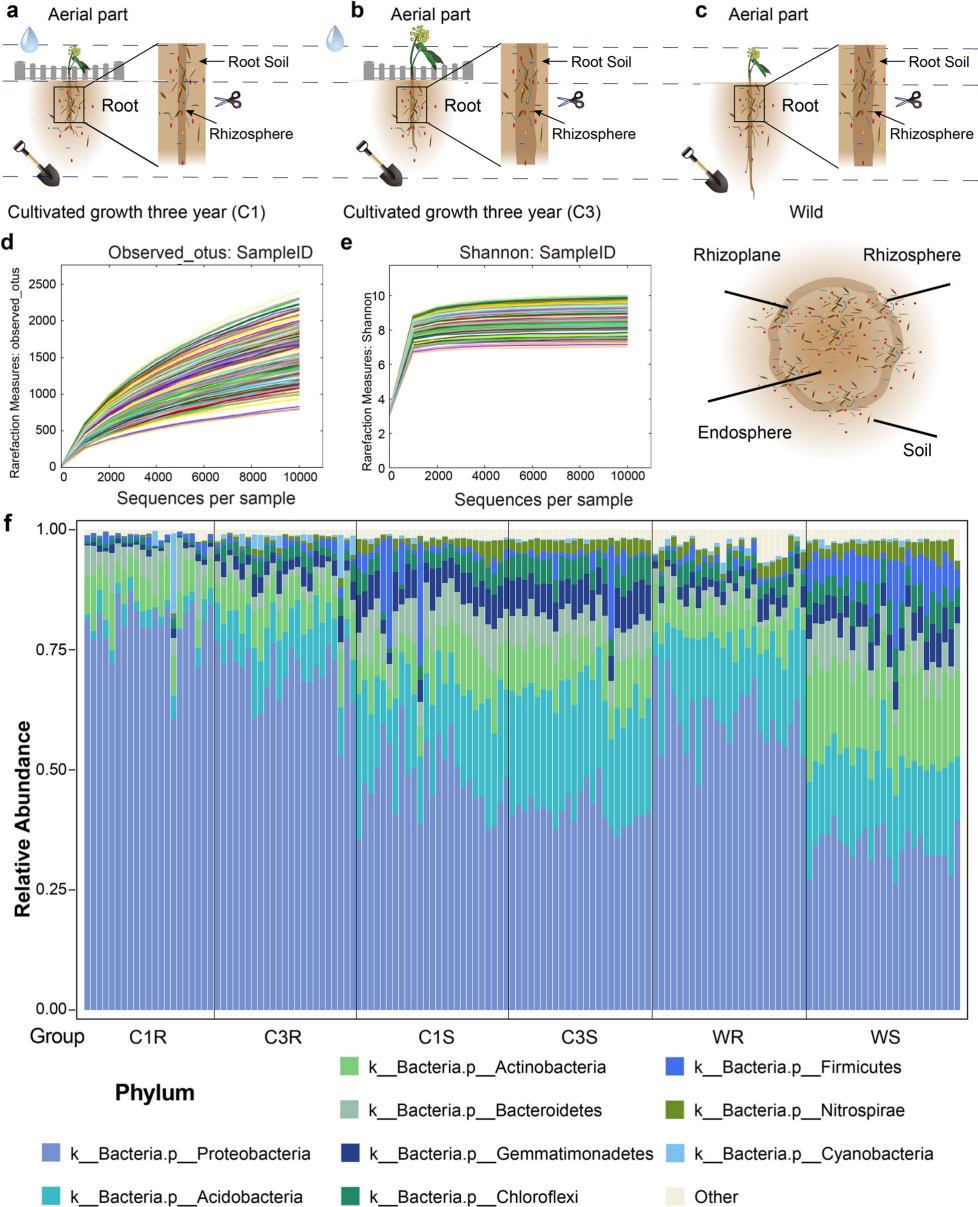
The microbial community compositions of samples from different group at phylum level. Sample collection schematic: **a** cultivation therophyte, **b** cultivation triennia, and **c** wild. 142 high quality 16 s rRNA sample data was acquired in this study. Spare curve to show the quantity and quality **d** Observed_otus, (**e**) Shannon index. **f** Here, except for the total relative abundance top 9 species, the others were clustered as “Other”. “C1R”, “C3R”, “C1S”, “C3S”, “WR”, “WS” in the figure represent “cultivated one-year growth *G. uralensis* rhizosphere”, “cultivated three-year growth *G. uralensis* rhizosphere”, “cultivated one-year growth *G. uralensis* soil”, “cultivated three-year growth *G. uralensis* soil”, “wild *G. uralensis* rhizosphere”, and “wild *G. uralensis* soil” successively

To explore the relationships among root-associated microbiota, the growth of *G. uralensis* and the accumulation of glycrrhizic acid as well as liquiritin, *G. uralensis* fresh root samples were collected for transcriptomics and metabolic study, and *G. uralensis* fresh root samples as well as soil samples were collected for microbial profiling. Based on the manual examination of medicinal plant by experts and local residents, about 3–5 years of natural wild *G. uralensis* was collected from village ShiJiQuan, Yanchi County, Ningxia province, China (106.861246^o^E, 37.98919^o^N). And this location represents authentic region for *G. uralensis* (Fig. 1a–c). The cultivated *G. uralensis* that grown for one year (C1) and three years (C3) were planted in two adjoining land, located in village TianJiZhang, Yanchi County, Wuzhong City, Ningxia province, China (107.267819^o^E, 37.814875^o^N), more than 5 miles far away from where wild *G. uralensis* samples were collected. In this study, we collected the *G. uralensis* root-associated rhizosphere microbiota (C1R, C3R) and soil microbiota (C1S, C3S, Additional file 1: Dataset 1).

After quality control, there were 30,268,250 high quality reads for 142 microbial samples (Additional file 1: Dataset 2, Table S1), on average each sample has 213,156 high-quality reads. And 7614 non-singletons OTUs were detected in this study. Spare curve (Fig. 1d, e) suggested that the adequate sequencing data was acquired for this research. The dominant bacteria in rhizosphere and soil microbiota are phylum Proteobacteria, Acidobacteria and Actinobacteria (Fig. 1f, the relative abundance (RA) table at phylum level is provided in Additional file 1: Dataset 3). The most abundant genera in the rhizosphere and soil microbiota are genus *Kaistobacter*, *Steroidobacter*, and *Rhodoplanes* of phylum Proteobacteria (Additional file 1: Figure S1, the RA table at genus level is provided in Additional file 1: Dataset 4).

### *G. uralensis* root assembles a rhizosphere microbiota which is different from that of the soil

**Fig. 2:**
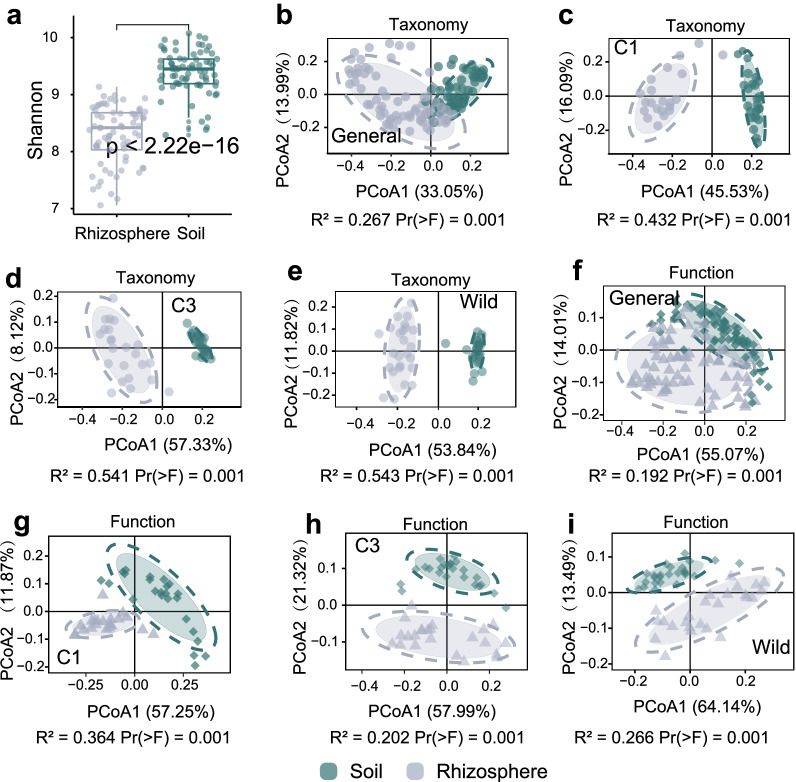
The different microbiota assembly patterns between rhizosphere and soil microbiota of *Glycyrrhiza uralensis* Fisch. **a** Alpha diversity comparison between rhizosphere and soil, based on Shannon index. Sample difference analysis based on the relative abundance at genus level with the “Jaccard” distance, **b** General, **c **Cultivated *G. uralensis* that are grown for one year (C1), **d** Cultivated *G. uralensis* that are grown for three years (C3), **e** Wild. Sample difference analysis based on the PICRUSt_predicted_functions with the “Jaccard” distance, **f** General, **g** C1, **h** C3, **i** Wild

Significant differences between the microbial diversity of the soil and the rhizosphere of *G. uralensis* (Fig. 2a–e and Fig. 1f, Additional file 1: Figure S1) were observed. And microbial alpha diversity was higher in the soil than that in rhizosphere (*P*-value < 0.01, Student Test, Fig. 2a).

We investigated the taxonomic distinctiveness of the rhizosphere and soil microbiomes of *G. uralensis*, and revealed clear differences in the microbial community structure between soil and rhizosphere of *G. uralensis* (Fig. 2b–e)*.* A phylum-level analysis of the communities revealed that rhizosphere and soil bacterial communities had distinct of relative abundance of the major groups (Fig. 1f). Principal coordinate analysis (PCoA) revealed that the functional composition of both wild and cultivated *G. uralensis’* rhizosphere was different from that of soil (Fig. 2f–i, the functional composition was provided in Additional file 1: Dataset 5). Multiple bacterial phyla, such as Acidobacteria and Actinobacteria, were present at a higher relative abundance in the rhizosphere, whereas proteobacteria was found higher abundance in the soil (Additional file 1: Figure S2), indicating that the *G. uralensis* root recruited the rhizosphere microbiota that was different from that of the soil with a different functional composition.

### Microbes colonize different *G. uralensis* with distinct patterns

**Fig. 3:**
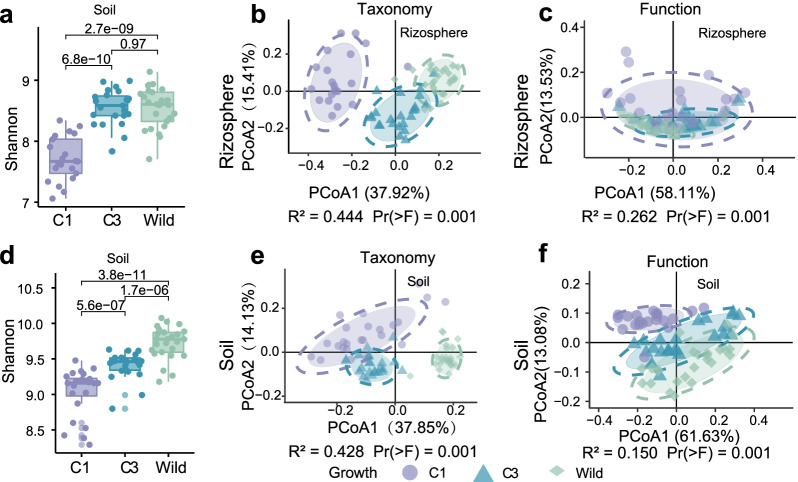
The association of root-associated microbiota assembly with growth of *G. uralensis.* **a** Alpha diversity comparison of rhizosphere microbiota of *G. uralensis* root with different growth years based on Shannon index. **b** Sample difference analysis of rhizosphere microbiota based on the relative abundance at genus level with the “Jaccard” distance. **c** Sample difference analysis of rhizosphere microbiota based on the PICRUSt_predicted_functions with the “Jaccard” distance. **d** Alpha diversity comparison of soil microbiota of *G. uralensis* root with different growth years based on Shannon index. **e** Sample difference analysis of soil microbiota based on the relative abundance at genus level with the “Jaccard” distance. **f** Sample difference analysis of soil microbiota based on the PICRUSt_predicted_functions with the “Jaccard” distance

**Fig. 4:**
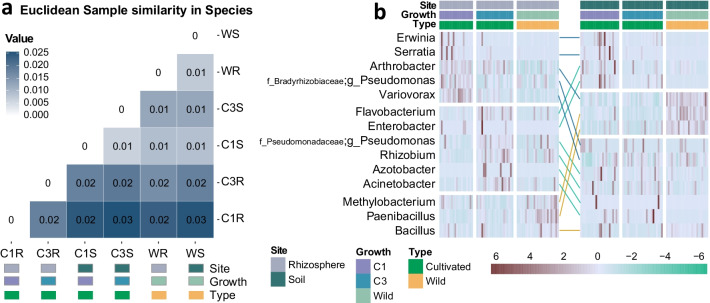
The assembly patterns of plant growth promoting rhizobacteria of *G. uralensis.* **a** Euclidean distance-based sample similarity at species level. And the annotation termed “C1R”, “C3R”, “WR”, represent “Rhizosphere microbiota of cultivated *G. uralensis* that are grown for one year”, “Rhizosphere microbiota of cultivated *G. uralensis* that are grown for three years”, “Rhizosphere microbiota of wild” rhizosphere separately; and “C1S”, “C3S”, “WS”, means “Soil microbiota of cultivated *G. uralensis* that are grown for one year”, “Soil microbiota of cultivated *G. uralensis* that are grown for three years”, and “Soil microbiota of wild *G. uralensis*”, successively. The “value” labeled in the figure is the Euclidean distance value. **b** The relative abundance distribution of the 14 previous reported plant growth promoting rhizobacteria (PGPR) that detected in the study

The composition of root microbial community composition appeared to be influenced by the types of *G. uralensis* (Fig. 3). The C3 *G. uralensis* had higher alpha diversity in the rhizosphere and soil microbial community than C1 *G. uralensis*, and the wild *G. uralensis* had higher alpha diversity in the rhizosphere and soil microbial community than the cultivated *G. uralensis* (Fig. 3a). A principal coordinate analysis (PCoA) based on the microbial community composition (Fig. 3b) and functional composition (Fig. 3c, Additional file 1: Dataset 5) from both rhizosphere and soil samples revealed a strong clustering in accordance with the type of *G. uralensis*. Microbial community assemblages significantly differed between wild and cultivated *G. uralensis*. As expected, the greatest differences in community assemblages, both rhizosphere and soil, were measured between wild and C1 *G. uralensis* (Fig. 4a). In comparison, the RA of *Pedomicrobium* and *Hyphomicrobium* were higher in the rhizosphere of wild *G. uralensis* than C1 and C3 (Additional file 1: Figure S2). In addition, the rhizosphere of C1 *G. uralensis* recruited a higher relative abundance of *Luteimona* and *Variovorax.* Compared with C1 *G. uralensis*, wild and C3 *G. uralensis* had a strong tendency to enrich taxa be capable of forming symbiotic nodule, such as *Rhizobium* (Fig. 4b and Additional file 1: Figure S2). When compared to the cultivation *G. uralensis*, the wild *G. uralensis* had a higher relative abundance of genus Candidatus *Nitrososphaera* in the rhizosphere (Additional file 1: Figure S2). These results suggested higher alpha diversity and complexity within the wild and C3 *G. uralensis* rhizosphere and soil microbiota than C1 *G. uralensis*.

The differences of rhizosphere microbiota of these different types of *G. uralensis* were more represented in PGPR and pathogens. As shown in Fig. 4b, the *Erwinia* is a genus of plant pathogens related to dry necrosis, and it was recovered at higher relative abundance in the C1 rhizosphere than that of C3 rhizosphere and wild rhizosphere. In addition, the genus *Rhizobium* was capable of forming symbiotic nodules on the roots were present at a higher relative abundance in the C3 rhizosphere. The genera *Bacillus* and *Methylobacterium*, which improves growth and nutrient uptake of plants, appear to be more abundant in the wild rhizosphere than C1 and C3.

### Core taxa of the *G. uralensis* rhizosphere microbiota

For each group of *G. uralensis*, distinct core microbiomes existed. 5, 17, 8 genera were identified in the core rhizosphere microbiota of wild, C1, and C3 *G. uralensis*, respectively (Additional file 1: Figure S3). In addition, 78 genera present in both core rhizosphere microbiota of wild, C1, and C3 *G. uralensis*. Multiple members affiliated with these core bacterial genera in both wild, C1, and C3 *G. uralensi*s, such as *Methylobacterium*, *Variovorax*, *Rhizobium* (Fig. 4b). However, the relative abundance of core rhizosphere microbiota of *G. uralensis* is influenced by sample type and growing time. In addition, plant–microbe interactions are very likely to be important factors that would influence the assembly of rhizosphere microbiota. Five core rhizosphere genera, *Saccharothrix*, *Phytohabitans*, *Hymenobacter*, *Lysinibacillus*, and *Cupriavidus*, were identified as unique core microbiota for wild *G. uralensis*. These five PGPRs were present in wild *G. uralensis*, indicating that wild *G. uralensis* has the ability to recruit these core PGPRs, which may not be found in cultivated *G. uralensis*. By contrast, there were 17 and 8 specific core rhizosphere microbes in C1 and C3 *G. uralensis*, respectively.

### Multi-omics profiling demonstrates a potential association between the growth of *G. uralensis* and the root-associated microbiota assemblage

**Fig. 5:**
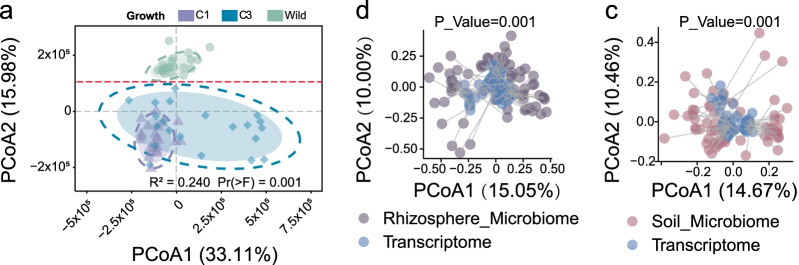
The sample similarity of transcriptomics in accordance with the *G. uralensis* root-associated microbiota. **a** The transcriptomics difference was performed between different group to figure out the influences of growth condition and growth year to the *G. uralensis.* FPKM (Fragments Per Kilobase of exon model per Million mapped fragments) table was the input table for difference analysis. And Euclidean method was used to calculate the sample similarity. At the bottom of each axis is the corresponding data distribution of different group. The red imaginary line in **a** distinguish the wild samples and cultivation samples well. Procrustes Analysis here was applied to correlation study of the *G. uralensis* root-associated microbiota and root transcriptomics. The dataset of microbial relative abundance table at genus level and the FPKM table were the input dataset for Procrustes Analysis. The scatter plots represent a significant agreement between the rhizosphere microbiota and transcriptomics (**b**), between soil microbiota and transcriptomics (**c**)

Rhizosphere microbial communities comprise a subset of colonists originating from the surrounding soil, and there was potential association between the growth of *G. uralensis* and the root-associated microbiota assemblage. We generated the transcriptomic profiles of the wild and cultivated *G. uralensis* (Fig. 5a), analyzed these profiles together with *G. uralensis*’ microbial profiles. We found that the rhizosphere microbial and transcriptomic profiles are in concordance based on the Manhattan-based microbiota distance and Manhattan-based transcriptomics distance (Fig. 5b, Monte Carlo *P-*value < 0.01). This finding was confirmed when test for soil microbiota variation and transcriptomics variation (Fig. 5c, Monte Carlo *P-*value < 0.01).

**Fig. 6:**
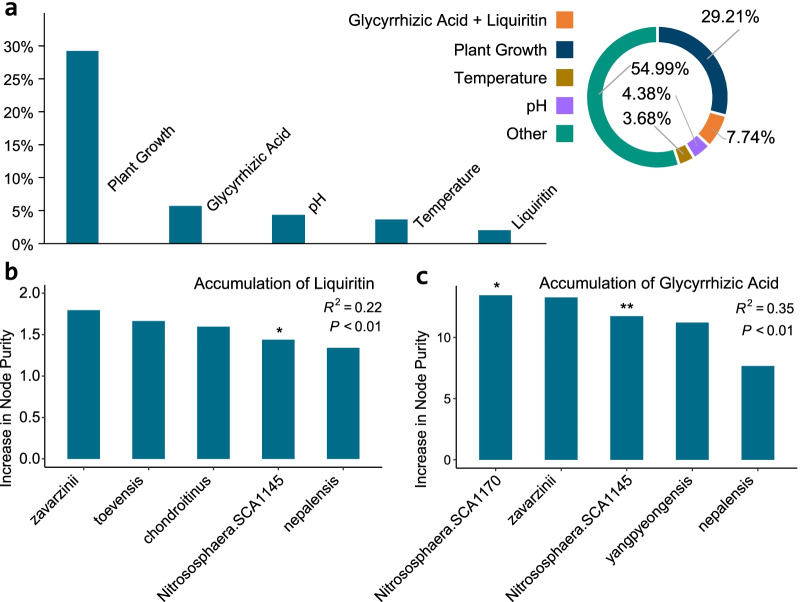
*G. uralensis* root-associated microbiota related to the accumulation of Glycyrrhizic acid and liquiritin. **a** The potential factors including plant growth year, glycyrrhizic acid and liquiritin accumulation, pH, soil temperature influences the *G. uralensis* rhizosphere microbiota in beta diversity. Random forest mean predictor importance (percentage of increase in node purity) of the five picked co-occurrence species for the accumulation of liquiritin (**b**) and glycyrrhizic acid (**c**). The accuracy importance measure was computed for each tree and averaged over the forest (1000 trees). Percentage of increase in node purity of variables were used to estimate the importance of these predictors, and higher percentage of increase in node purity imply more important predictors. Significance levels are as follows: *P < 0.05 and **P < 0.01

The beta diversity of the *G. uralensis* root-associated microbiota can be influenced by multiple factors, including the soil pH and temperature, the liquiritin and glycyrrhizic acid content (Fig. 6a). Our Random Forest regression analysis showed that the accumulation of secondary metabolites of the *G. uralensis* was related to the rhizosphere microbiota, as evidence by the strong correlation between the liquiritin content of *G. uralensis* and rhizosphere microbiota (Fig. 6b, Joint hypotheses test, *P*-value < 0.01), as well as between the glycyrrhizic acid content and rhizosphere microbiota (Fig. 6c, Joint hypotheses test, *P*-value < 0.01). In addition, our analysis showed that beta diversity at genus level among the C1, C3 and wild rhizosphere can be explained by the plant growth year with 29.21%, accumulation of glycyrrhizic acid and liquiritin with 7.74%, pH with 4.38%, temperature with 3.68%, and 54.99% unexplained (Fig. 6a). And the beta diversity at species level can be explained by plant growth year with 8.12%, the accumulation of glycyrrhizic and liquiritin with 3.34%. Additionally, the beta diversity of *G. uralensis* soil at genus level can be explained by plant growth year with 8.20%, the accumulation of glycyrrhizic and liquiritin with 3.4%, and the temperature with 12.31%, pH with 3.02%, and 73.07% unexplained (Additional file 1: Figure S4).

### A predictive model that links *G. uralensis* root-associated microbiota with growth status and accumulation of secondary metabolites

**Fig. 7:**
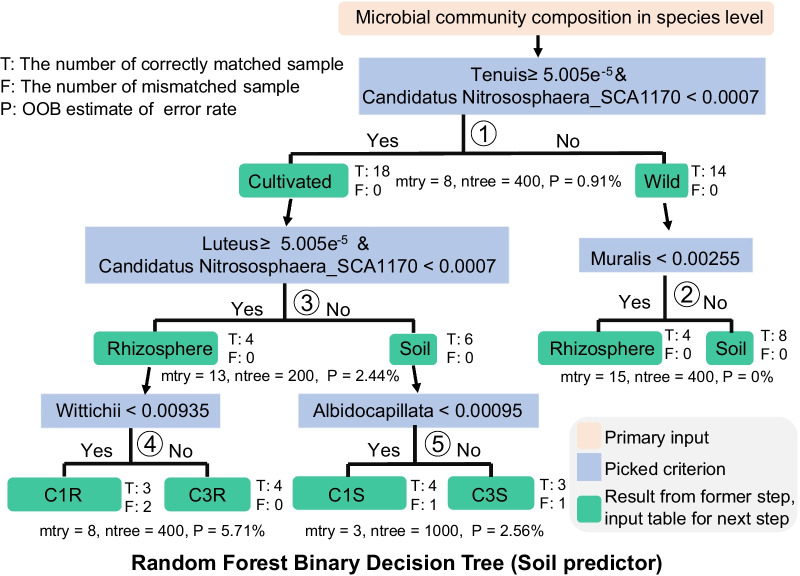
The plant growth condition and growth year were associated with the root-associated microbial community composition in *G. uralensis.* The primary input data for the building of a binary decision tree named soil predictor in this study is root-associated microbial community composition at species level. In every step of this soil predictor (①–⑤), the relative abundance table was normalized into a co-occurrence species table, with every species present more than 50% samples of no less than one small group. And, 80% of the samples were randomly chosen as training dataset and the rest 20% samples used as the validation dataset. Annotations: T: The number of correctly matched samples in the validation test. F: The number of mismatched samples in the validation test. P: OOB (out-of-bag) estimate of the error rate of the built prediction model. mtry: Number of variables randomly sampled as candidates at each split. ntree: Number of trees to grow

The prediction model identified a strong association among *G. uralensis*’ root-associated microbiota, environmental factors, growth status and accumulation of secondary metabolites (Fig. 7). Here a Random Forest Binary Decision Tree called soil predictor was established based on root-associated microbiota. Area under the curve (AUC) of receiver operating characteristic curve (ROC) was used to evaluate the accuracy of this soil predictor. Many bacterial genera were strongly associated with liquiritin or glycyrrhizic acid, despite significant differences in the relative abundance of the three types of *G. uralensis.* For instance, RA of *Tenuis* ≥ 0.15 × 10^–3^ (AUC = 0.97, Additional file 1: Figure S5) and RA of Candidatus *Nitrososphaera*_*SCA1170* < 0.70 × 10^–3^ (AUC = 0.96, Additional file 1: Figure S5) differentiated the microbial sample belongs to wild and cultivated *G. uralensis*, which have different accumulation of liquiritin and glycyrrhizic acid. In addition, RA of *Obscurus* < 0.25 × 10^–2^ (AUC = 0.99, Additional file 1: Figure S6) differentiated the rhizosphere and soil samples of wild *G. uralensis*. Besides, RA of *Amycolatopsis Thermoflava* ≥ 0.25 × 10^–3^ (AUC = 0.97, Additional file 1: Figure S7) differentiated the rhizosphere and soil samples of cultivated *G. uralensis*. Moreover, RA of *Legionella Quinlivanii* < 0.94 × 10^–2^ (AUC = 0.91, Additional file 1: Figure S8) differentiated the C1 and C3 *G. uralensis* using rhizosphere samples, with an error rate of 22.22%. And the RA of *Albidocapillata* < 0.95 × 10^–3^ (AUC = 0.91, Additional file 1: Figure S9) differentiated the C1 and C3 *G. uralensis* using soil samples, with an accuracy of 77.78%. Taken together, these findings support the existence of strong relationships between the *G. uralensis* root-associated microbiota assemblage, the growth status, and the accumulation of the liquiritin and glycyrrhizic acid.

## Discussion

The root-associated microbiota's association with environmental factors, growth status and accumulation of secondary metabolites, remain unclear, rendering the patterns behind the multi-omics regulations for medicinal *G. uralensis* elusive. In this study, we performed a comprehensive study of the taxonomic features of wild and cultivated *G. uralensis* rhizosphere microbiota to better determine root-associated microbiota and metabolite-plant-microbes properties in this habitat.

### The assemblage of *G. uralensis* root-associated microbiota

The plant growth condition and growth year are two of the most important factors influencing the accumulation of secondary metabolites[33, 34] and root-microbiota assemblage[35–37]. In our study, the plant growth and growth conditions of *G. uralensis* were found to be strongly related to the assemblage of the root-associated microbiota. *G. uralensis* of different growth years had specific rhizosphere and soil microbiota (Fig. 3b): proteobacteria was enriched in the C1 *G. uralensis* rhizosphere and soil, while acidobacteria was enriched in the C3 *G. uralensis* rhizosphere and soil. The similar results were found to the root associated fungi. As previously reported, the diversity and richness of endophytic fungi and arbuscular mycorrhizal fungi was affected by the growth period of *Glycyrrhiza* significantly [38]. Furthermore, the microbiota of wild and cultivated *G. uralensis* differed, a finding that has previously been reported [39]. The genus *Kaistoabacter,* which is commonly active or applied in land restoration of polycyclic aromatic hydrocarbon cadmium (PAH-Cd) co-contaminated soil [40], was enriched in the wild *G. uralensis*. And the *Novosphingobium*, contains many genes encoding PAH and components involved in xenobiotic degradation [41], like *acidiphillum*, *capsulatum*, and *nitrogenifigens*, was also enriched in the wild *G. uralensis*. The enrichment of the microbes in the rhizosphere can be attributed to plant lifestyles [42]*.* The assemblage of root-associated microbiota, especially genus *Kaistoabacter* and *Novosphingobium*, was likely to be the result of a living strategy in response to *G. uralensis* root exudations such as liquiritin, isoliquiritigenin, dimethyl phthalate, diethyl phthalate.

The particular microbial taxa recruited to the rhizosphere from the soil microbial reservoir vary among *G. uralensis* from different growth status, especially the PGPR, including *Pseudomonas*, *Azospirillium, Azotobacter*, *Bacillus*, *Burkholderia*, *Enterobacter*, *Rhizobium*, *Flavobacterium*, *Methylobacterium*, *Serratia* and *Mesorhizobium*. Most of these PGPRs in *G. uralensis* have been reported to secrete amount of phytohormones, such as IAA, auxins, cytokinin and abscisic acid, to promote plant growth and nutrient cycling with the soil [43]. In addition, Kanosamine, oligomycin A, xanthobaccin and zwittermicin produced by *Bacillus* have been identified as antibiotics that have antibacterial, antifungal, antiviral, antihelminthic, antimicrobial, cytotoxic, phytotoxic, antioxidant, and antitumor properties. In our research, the enrichment of *Bacillus* in the roots of *G. uralensis* might enhance its resistance to pathogen to adapt to the growth conditions. These results informed potential relationship between root-associated microbiota assemblage and the growth of *G. uralensis*, and even the accumulation of the medicinal components.

### The core root-associated microbiota of *G. uralensis*

Different types of *G. uralensis* apparently selected a particular core microbiome. The core microbiota of the plants contributes to plant growth and health [42]. We discovered that some of these core rhizosphere microbes were specific to different types of *G. uralensis*. However, some of the core rhizosphere microbes identified in the wild *G. uralensis* overlap with those identified in C1 and C3 *G. uralensis*, suggesting that many plant factors driving community assembly may be common between different types of *G. uralensis*. Furthermore, some of these core root-associated microbes have been reported to be PGPR, such as *Methylobacterium*, *Variovorax*, *Rhizobium*, *Saccharothrix*. These microorganisms are likely to be important related bacteria for the growth of *G. uralensis*. The genus *Saccharothrix* represents a group of non-mycorrhizal PGPR, belonging to gram-positive actinomycetes with branching vegetative mycelium *Lysinibacillus*. The genus *Saccharothrix* was found to be a unique core microbe of wild *G. uralensis*, can synthesizes indole via sodium succinate to promote plant growth. Another unique core microbe of wild *G. uralensis*, *Cupriavidus* has the ability to regulate the ethylene level in legumes. These unique core microbes of wild *G. uralensis* are likely to promote the metabolism of wild *G. uralensis* secondary metabolites.

The identification of a core of rhizosphere microbiota for different types of *G. uralensis* provides a useful starting point for future studies that could exploit synthetic communities to determine the interaction between microbes in their interactions with *G. uralensis*.

### Correlations between the assemblage of root-associated microbiota and accumulation of secondary metabolites

Our transcriptomic study of the *G. uralensis* root, together with root-associated microbial profile, proved that transcriptomic and microbial profiles for *G. uralensis* are largely concordant, whereas the wild *G. uralensis* was apparently dissimilar to the cultivated in terms of both transcriptomic and microbial profiles. However, no statistically significant correlations were found between the expressions of key genes in the glycyrrhizic acid or liquiritin biosynthesis pathways, and RA of root-associated microbiota, which could be explained by the delay effects of the PGPR to the *G. uralensis* root.

**Fig. 8:**
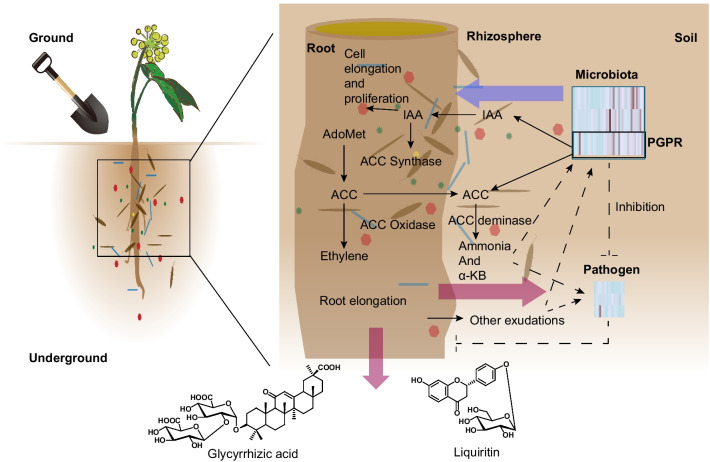
Schematic model of the *G. uralensis* root-associated microbiota, with special focus on the influence of PGPR on the plant growth and the accumulation of glycyrrhizic acid and liquiritin. PGPR was reported to act as additional function biome to the host plant, especially the extra heteroauxin (eg: IAA) supply to the host plant. The arrows indicate a physical or chemical step in the mechanism. The symbol “⊥” means ethylene inhibit root elongation. The dotted arrow indicate a hypothetical physical step, and the symbol “⊥” in dotted line means hypothetical inhibition of the pathogen to *G. uralensis*. The thick arrow to the left means the deduced influence from the root-associated microbiota to the G. uralensis root. The thick arrow to the right means the deduced influence from the G. uralensis root to the root-associated microbiota. The thick arrow pointing down means the deduced influence from the *G. uralensis* root to the root-associated microbiota. Key: IAA, indoleacetic acid; ACC, 1-aminocyclopropane-1-carboxylic acid; AdoMet, S-adenosyl-methionine, α-kB, α-ketobutyrate

Furthermore, environmental factors such as drought, pH, and temperature have a significant impact on the microbiota associated with roots [12–14]. This investigation found a correlation between the accumulation of liquiritin and glycyrrhizic acid and enrichment of genus Candidatus *Nitrososphaera* in the rhizosphere of wild *G. uralensis* (Additional file 1: Figure S2, Pearson correlation: glycyrrhizic acid, *R*^2^ = *0.51*, *P* < 0.01; liquiritin, *R*^2^ = *0.22*, *P* = 0.09). Candidatus *Nitrososphaera* has been reported to be an ammonia-oxidizing genus that enhances the accessible nitrogen in the soil, and promotes the production through the promotion of plant growth [44]. We deduced that there are potential molecular mechanisms of the root-associated microbiota interact with *G. uralensis* (Fig. 8). On the one hand, the root-associated microbiota influences the plant growth and the accumulation of secondary metabolites [38], including glycyrrhizic acid and liquiritin, as well as the adaptability of *G. uralensis* to the environments. For example, the PGPR produced indoleacetic acid (IAA) to promote the growth of host [45]. And on the other hand, these interactions would have an impact on the assemblage of the *G. uralensis* root-associate microbiota. Previous studies have proved that the quality or quantity of cultivated *G. uralensis* is lower than that of wild *G. uralensis*, especially the contents of flavonoid glycosides and triterpenoid saponins [22]. One possible explanation for this difference is that the secondary metabolism in wild *G. uralensis* is more active. Besides, the underlying causes of the difference accumulation of secondary metabolites would be culture methods and growth environment, and the root-associated microbiota mediated the effects of the environmental factors and culture methods [38]. Manipulation of root-associated microbiota composition during cultivation would thus be extremely beneficial for increasing *G. uralensis* growth as well as liquiritin and glycrrhizic acid production. Future experiments to verify the promoting effect of the relevant microbial community composition could include inoculating wild *G. uralensis* root microorganisms into cultivated *G. uralensis*, observing the metabolism of root exudates, and monitoring root microorganism changes during the growth process; testing wild and cultivated *G. uralensis* root exudates under sterile conditions to verify secondary metabolic differences caused by differences in microbial enrichment in the root.

Furthermore, we performed a predictive model based on *G. uralensis* root-associated microbiota, which highlighted a strong link between *G. uralensis* root-associated microbiota and growth status, and accumulation of secondary metabolites, presented compelling evidence to the relationship among the concert effects of *G. uralensis* growth status, secondary metabolites, and the root-associated microbiota assemblage (Fig. 7). These results could lead to a predictive model for better understanding of *G. uralensis’* growth status and accumulation of secondary metabolites. Based on these findings, further research could deepen our knowledge on how the *G. uralensis* root assembles a high-efficiency root-associated microbiota under multiple environmental stresses, resulting in a higher yield of glycyrrhizic acid and liquiritin.

## Conclusion

The *G. uralensis* root's rhizosphere microbiota differed from soil microbiota in terms of alpha and beta diversity, as well as the microbial community functional composition. The growth status, especially the plant growth year, of *G. uralensis*, as well as the accumulation of glycyrrhizic acid and liquiritin, were found to be strongly correlated to the structure and function of root-associated microbiota. Additionally, the assemblage of PGPR in the *G. uralensis* root was also associated with plant growth time and growth conditions. Furthermore, the microbial community composition in both rhizosphere microbiota and soil microbiota were found to be closely related to *G. uralensis*'s gene expression. Finally, the predictive model emphasized the relationships among *G. uralensis* root-associated microbiota, growth status, and accumulation of the liquiritin and glycyrrhizic acid. These findings shed light on how *G. uralensis* root interacts with root-associated microbiota, as well as how accumulation levels of glycyrrhizic acid and liquiritin associate with root-associated microbiota. To our knowledge, this is one of the pioneer studies to explore root-associated microbiota of *G. uralensis* in different growth years and different growth conditions, and to combine multi-omics data to study the concert effect of growth status and the assemblage of the root-associated microbiota on the accumulation of secondary metabolites. The results confirmed that the root-associated microbial communities of *G. uralensis* played important roles in its growth as well as the accumulation of the glycyrrhizic acid and liquiritin, suggesting that optimizing the root-associated microbial communities could lead to better cultivation of *G. uralensis*. This study advanced our mechanistic understanding of how shifts in microbial community composition mediate and reflect the effects of plant secondary metabolites accumulation, especially liquiritin and glycyrrhizic acid, in medicinal plant *G. uralensis*.

## Supplementary Information

**Additional file 1**. Supplementary data.

## Data Availability

The root-associated microbiota 16S rRNA sequencing raw data was deposited to NCBI’s Sequence Read Archive (SRA) database under the BioProject number PRJNA705567. And the *G. uralensis* root transcriptomics raw sequencing data was deposited to NCBI’s Sequence Read Archive (SRA) database under the BioProject number PRJNA705545.

## Abbreviations

RA: Relative abundance
PCoA: Principal coordinates analysis
LDA: Linear discriminant analysis
PAHs: Polycyclic aromatic hydrocarbon
IAA: Indole-3-acetic acid
PGPR: Plant growth-promoting rhizobacteria
ACC: 1-Aminocyclopropane-1-carboxy-late
FPKM: Fragments per kilobase of exon model per Million mapped fragments
PERMANOVA: Permutational multivariate analysis of variance
HPLC: High performance liquid chromatography
C1R: Rhizosphere microbiota of the cultivated *G. uralensis* that are grown for one year
C3R: Rhizosphere microbiota of the cultivated *G. uralensis* that are grown for three years
C1S: Soil microbiota of the cultivated *G. uralensis* that are grown for one year
C3S: Soil microbiota of the cultivated *G. uralensis* that are grown for three years
WR: Rhizosphere microbiota of wild *G. uralensis*
WS: Soil microbiota of wild *G. uralensis*
RIN: RNA integrity number
RT-PCR: Reverse transcription-polymerase chain reaction
rDNA: Ribosomal DNA
α-kB: α-Ketobutyrate
Soilpredictor: Random Forest Binary Decision Tree
ROC: Receiver operating characteristic curve
VPA: Variance Partitioning Analysis
OBB: Out-of-bag estimate of the error rate
