# Supplementary material for: Glycyrrhiza uralensis Fisch. Root-associated microbiota: the multifaceted hubs associated with environmental factors, growth status and accumulation of secondary metabolites
Source: Environ Microbiome. 2022 May 7;17:23. doi: 10.1186/s40793-022-00418-0 (PMC9080174; doi:10.1186/s40793-022-00418-0)
Supplement: Supplementary file 1 — Additional file 1. Supplementary data. [file 40793_2022_418_MOESM1_ESM.docx]

***Supplementary Information***

**Content**

Methods 3

**Biomarker Analysis** 3

**Transcriptomic data collection and analysis** 3

RNA extraction, processing and normalization 3

Date processing and differential variability analysis 3

**Metabolic data collection analysis** 4

Glycyrrhizic acid and liquiritin extraction 4

Reagents and biomarkers preparation 4

High performance liquid chromatography instrumentation and chromatography analysis 5

**Statistics methods** 5

Euclidean distance calculation 5

Permutation ANOVA 5

**Function prediction** 5

**Random Forest** 6

Supplementary Table 7

Table S1 7

Supplementary Figures 8

Supplementary Figure 1. The microbial community composition of different group in genus level 11

Supplementary Figure 2. Relative abundance distribution of biomarkers of the G. uralensis root-associated rhizosphere and soil microbiota 12

Supplementary Figure 3. Core taxa of the *G. uralensis* rhizosphere microbiota 13

Supplementary Figure 4. Relationships of G. uralensis root soil microbiota and the accumulation of Glycyrrhizic acid as well as liquiritin 14

Supplementary Figure 5. Prediction of the plant growth condition based on root-associated microbial community composition 15

Supplementary Figure 6. Prediction of the origin site of wild G. uralensis based on the root-associated microbial community composition 16

Supplementary Figure 7. Prediction of the origin site of cultivated G. uralensis based on the root-associated microbial community composition 17

Supplementary Figure 8. Prediction of the growth year of cultivated *G. uralensis* based on the rhizosphere microbiota microbial community composition 18

Supplementary Figure 9. Prediction of the growth year of cultivated *G. uralensis* based on soil microbial community composition 19

References 20

# Methods

### Biomarker Analysis

To figure out the potential biomarkers of vital importance in forming special status in the interactions between microbiota and *G. uralensis*, especially the accumulation of glycyrrhizic acid and liquiritin, LEfSe online tools was applied to the microbial RA table. For this purpose, the relative table of phylum, class, order, family, genus and species level were joined into a general table. And then, the taxa unassigned lines were removed before input to the LEfSe pipeline. The alpha value for the factorial Kruskal-Wallis test among classes and pairwise Wilcoxon test between subclasses were set to 0.05, and the logarithmic LDA score for discriminative features was set into 3.5 in this study.

## Transcriptomic data collection and analysis

### RNA extraction, processing and normalization

The total RNA was extracted from the freeze *G. uralensis* root with OmniPlant RNA Kit (DNase I) (CWBIO, China) in accordance with the kit instructions. And to acquire high quality total RNA, the fresh root sample freeze with liquid nitrogen describe in the former section of sample collection, and before RNA extraction the root was grinded into powder, and in the grinding process moderate liquid nitrogen was added for cooling. The total RNA then, was checked with Nanodrop 2000 of the concentration and purity. The integrity analysis of RNA fragment was performed with agarose gel electrophoresis, and the RNA integrity number (RIN) was calculated with Agilent2100 system. The quantity of RNA template for sequencing library building was 5 µg in the concentration of no less than 200 ng/µL and OD260/280 between 1.8 to 2.2. The mRNA was enriched through magnetic beads with Oligo(dT) capture to pairing the ployA of the total RNA. And the mRNA was randomly cut into small fragments about 200 bp. The template mRNA was replicate into cDNA with reverse transcriptase through reverse transcription-polymerase chain reaction (RT-PCR). And the sequencing process was performed with IlluminaHiseq platform.

### Date processing and differential variability analysis

To acquiring highly quality reads for following analysis, Trimmomatic in version v0.38 was applied to the raw reads with ambiguous nucleotides rate more than 10%, and the quality value over 20, length longer than 50 bp. Using HISAT2 (v2-2.1.0) with default parameters, the clean reads were aligned to the genome (downloading at http://ngs-data-archive.psc.riken.jp/Gur-genome/download.pl). To assemble the transcripts, the StringTie (v1.3.6) was with the following options: -f 0.3 -j 3 -c 5 -g 100. With default parameters, the assemblies were then compared with the reference genome by gffcompare (v0.11.2). And the expression levels of genes were calculated using StringTie with -e -A parameters. Besides, the read count information was extracted using the prepDE.py script. Differential gene expression analysis was performed with package DESeq2, and the 2-fold change adjust p-value was set to 0.05. Using online BlastKOALA tool, the reads were annotated by the Kyoto Encyclopedia of Genes and Genomes (KEGG) databases. The functional enrichment analysis of DEGs was analyzed by software KOBAS. The threshold adjusted p-value for functional enrichment analysis was set to 0.05.

## Metabolic data collection analysis

### Glycyrrhizic acid and liquiritin extraction

Focus on the accumulation of liquiritin and glycyrrhizic acid, here the metabolic content was measured with the high-performance liquid chromatography (HPLC). Firstly, the natural dried root fragments of *G. uralensis* were sliced into 2 mm thin, and then smashed into powder. Secondly, filter the powder with a 180 µm sieve. Thirdly, 0.2 g filtered powder for every sample was dissolved into a 100 ml volumetric flask with 70% alcohol, and ultrasonic treat (250 W, 40 kHz) for 30 min. And then, when the temperature of suspension falls into the room temperature, add extra 70% alcohol into 100 ml followed with fully blending. After that, the mixture was filtered with a 0.45 µm filter for the HPLC experiment.

### Reagents and biomarkers preparation

The reagents and biomarkers were as follows: acetonitrile in chromatographic (TEDIA, USA); trifluoroacetic acid, liquiritin (93.1%) and glycyrrhizic acid (97%) were bought from National Institutes for Food and Drug Control (China). The solvent is 70% alcohol. 4.01mg liquiritin (93.1%) was dissolved into 100 ml and 9.99 mg glycyrrhizic acid (97%) was dissolved into 25 ml. The two biomarkers suspension was then mixed into HPLC biomarker in 1:1 of volume, which the final concentration of the liquiritin would be 20.1 μg/mL and the glycyrrhizic acid 195.74 μg/mL.

### High performance liquid chromatography instrumentation and chromatography analysis

Instrument Type: Dionex U3000 HPLC System (Germany)

Software: chameleon

Flow rate: 1.0 mL/min

Detector: DAD-3000 and wavelength: 237 nm

Run time: 30 min

Injection volume: 5 μL

Column dimensions: Ultimate C_18_, 150 mm × 4.6 mm LD, 5 μm

## Statistics methods

**Euclidean distance calculation**

Euclidean distance was used for calculating the similarities of samples (**Figure 4 a**) in this study. R script ‘dist’ function was applied to generate the Euclidean distance. Method parameter was set as ‘eulidean’. The relative abundances of microbial community in wild *G. uralensis* soil was set as the reference. The detail steps were described in our former study [1].

**Permutational multivariate ANOVA**

Depicting the differences among groups (**Table 1)**, and significant test of the different distribution in PCoA analysis, PERMANOVA test was run on R command ‘adonis2’ in library ‘vegan’. The test data was transformed into a 2-dimension matrix, with each column presenting the detected values for each variable and each row presenting each detection of each sample. The ‘environment’ of the test was also input as a 2-dimension matrix with the same row title and each column presenting different grouping information. While running the script, the column name was assigned as ‘environment’ and method was assigned as ‘jaccard’, and permutations times was set to 999.

**Function predication**

Predictive functional profiling of campus microbial communities was generated by PICRUSt 1.1.0 from 16S rRNA marker genes [2], which allows the prediction of functional pathways from the 16S rRNA reads. First, a collection of closed-reference OTUs was obtained by using QIIME ‘pick_closed_reference_otus.py’ script in default settings. Then the script ‘normalize_by_copy_number.py’ was applied to normalize the output table based on the predicted 16S rRNA copy number. Final functional predictions, inferred from the metagenomes, were generated with the script ‘predict_metagenomes.py’.

**Random Forest**

A binary decision tree was built in this study named soil predictor. For the plant growth condition, and plant growth year as well as the accumulation of liquiritin and glycyrrhizic acid prediction, the building of soil predictor was based on *G. uralensis* root-associated microbiota microbial community composition. The training data for Random Forest is root-associated microbiota microbial community composition in species level. In every step (①-⑤) (**Figure 7**), the relative abundance table was normalized into a co-occurrence species table, with every species present more than 50% sample of no less than one small group. And, 80% of the samples were randomly chosen as training dataset and the rest 20% samples used as the validation dataset. The soil predictor was built with package “randomForest” [3] in R language, and valuated with “pROC” [4].

# Supplementary Table

**Table S1. Sample name used for sample collection.** The samples were collected in august, 30-31, 2018.

| Sample | C1 | C3 | Wild |
| --- | --- | --- | --- |
| Rhizosphere microbiota samples | 21 | 24 | 25 |
| Soil microbiota samples | 23 | 24 | 25 |
| Transcriptomics samples | 20 | 23 | 21 |
| Metabolic samples | 25 | 25 | 25 |

# Supplementary Figure


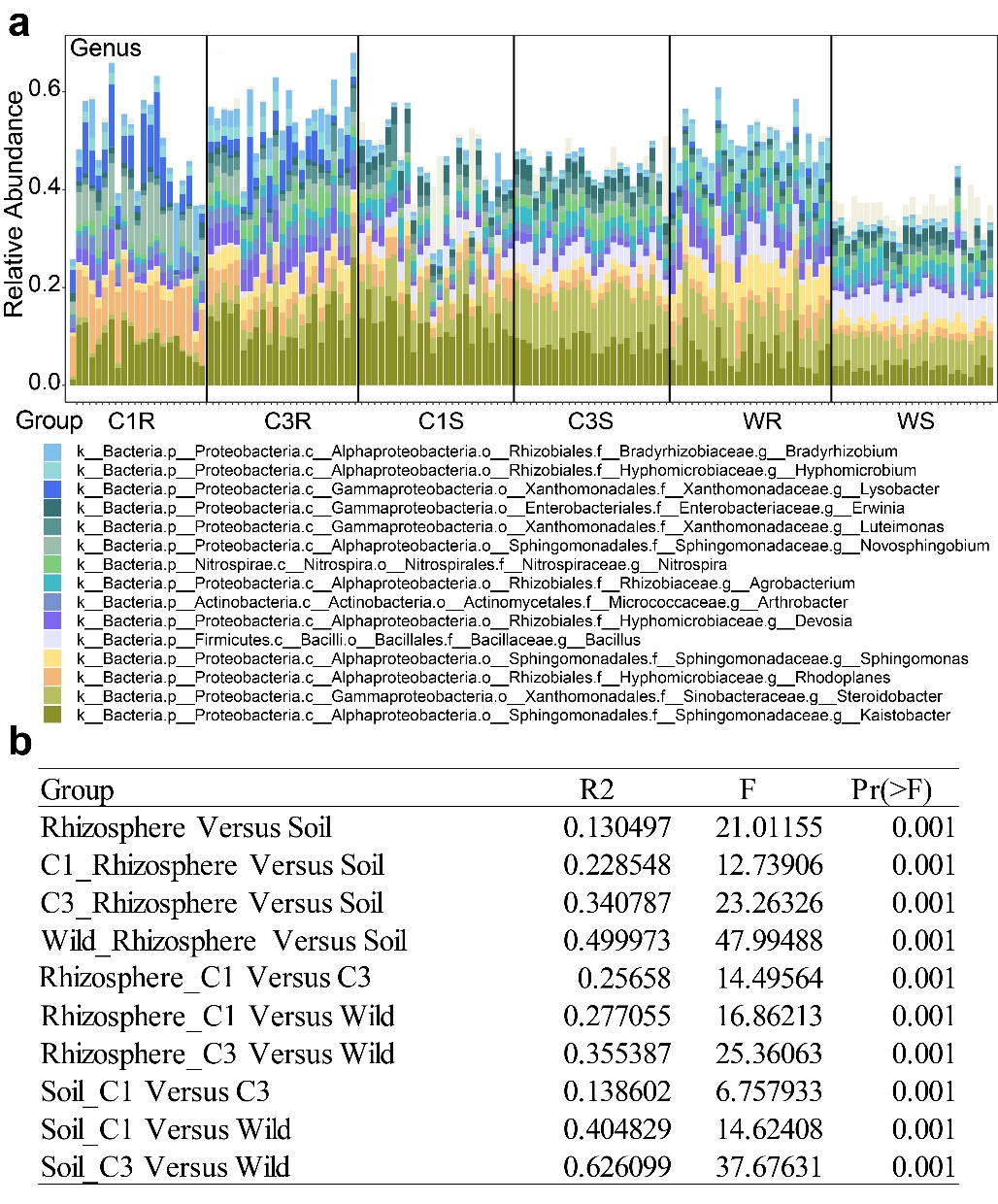


**Figure S1. The microbial community composition of different group in genus level.** (**a**) Here, the total relative abundance top 16 species, was plotted. (**b**) Permanova analysis to test the sample difference between different group in genus relative abundance table. The permutation times was set to 999.


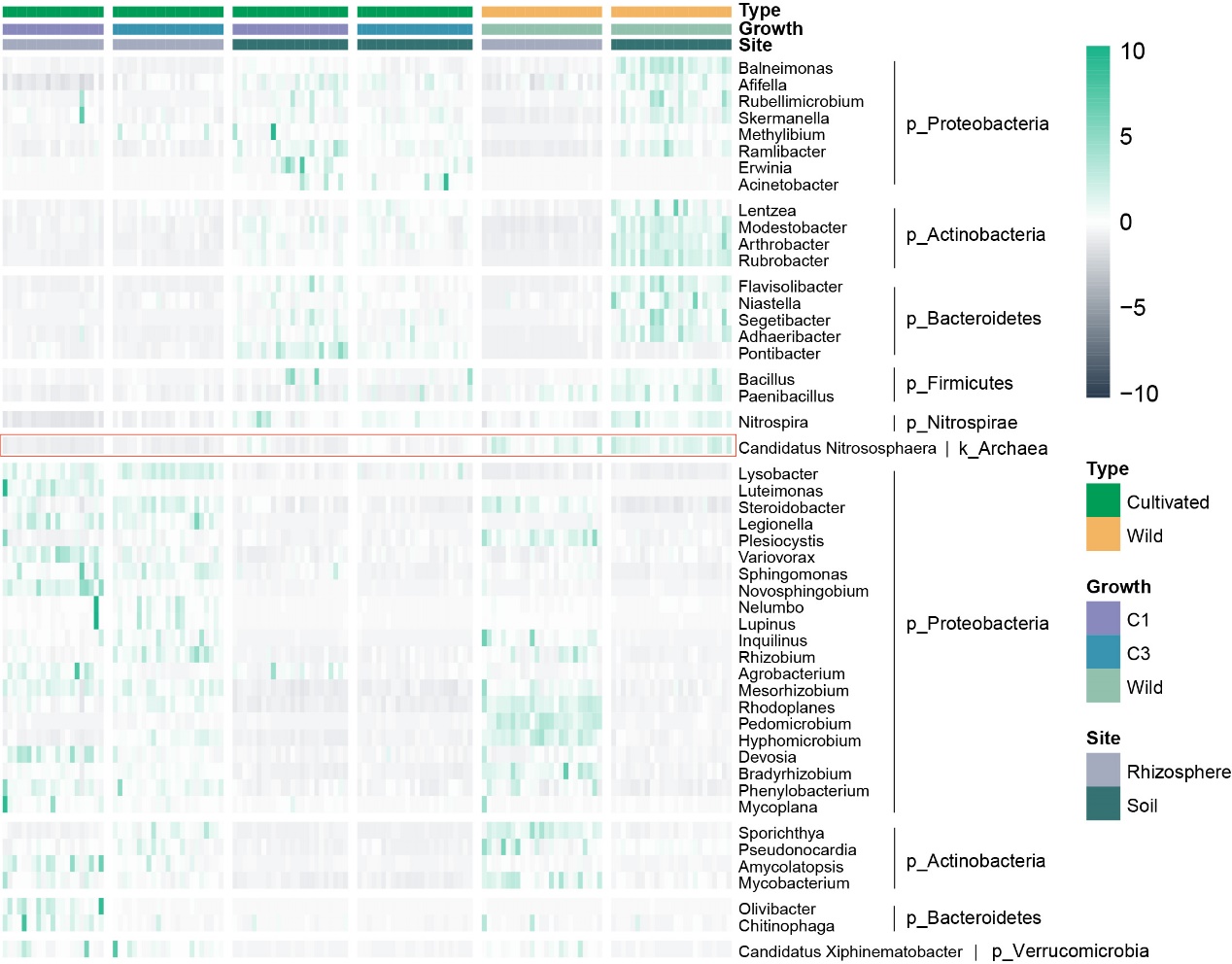


**Figure S2. Relative abundance distribution of biomarkers of the *G. uralensis* root-associated rhizosphere and soil microbiota.** Threshold of the logarithmic LDA score for discriminative features picking was set to 3.5.


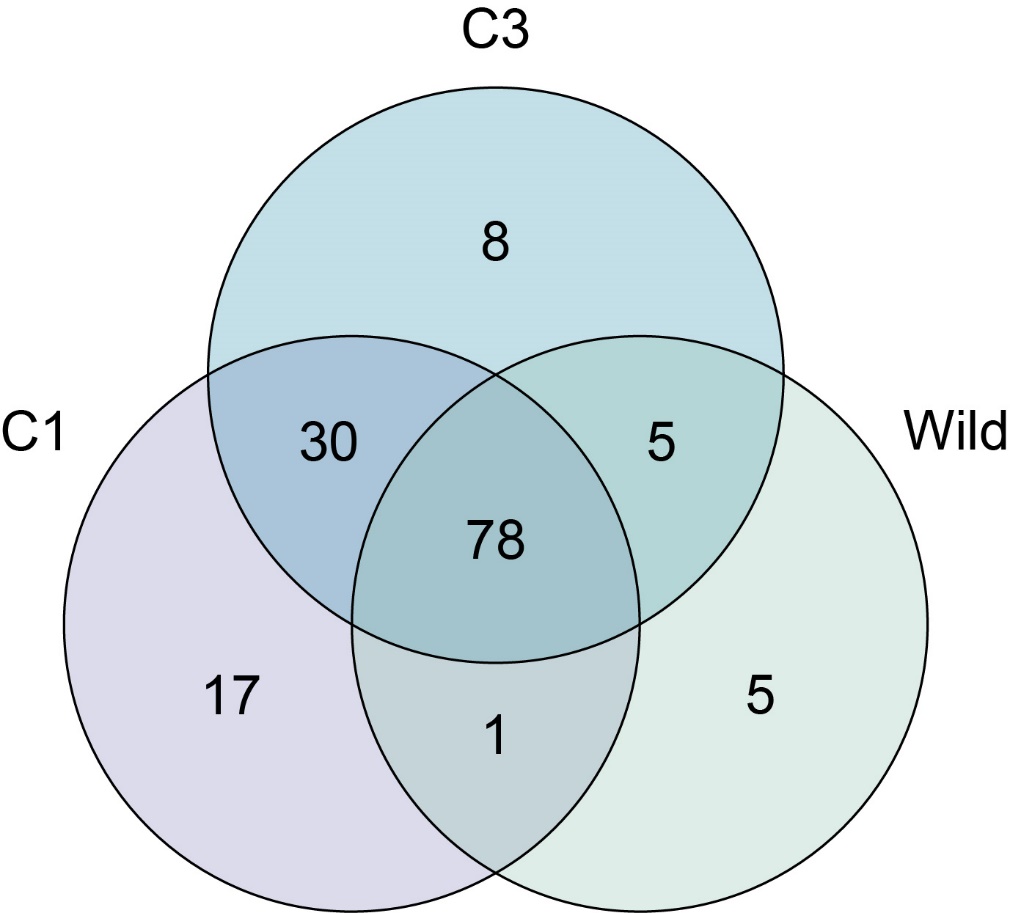


**Figure S3. Core taxa of the *G. uralensis* rhizosphere microbiota.** The definition of the core taxa is present in more than 50% samples of the specify genus.


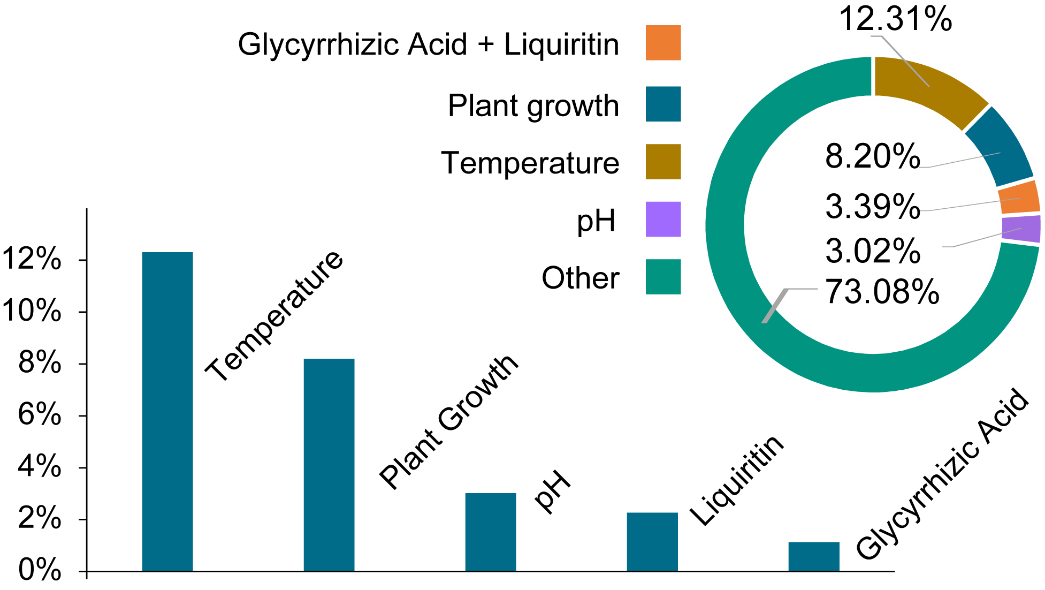


**Figure S4.** **Relationships of *G. uralensis* root soil microbiota and the accumulation of Glycyrrhizic acid as well as liquiritin.** The potential factors including plant growth year, glycyrrhizic acid and liquiritin accumulation, pH, soil temperature influences the soil microbiota in beta diversity.


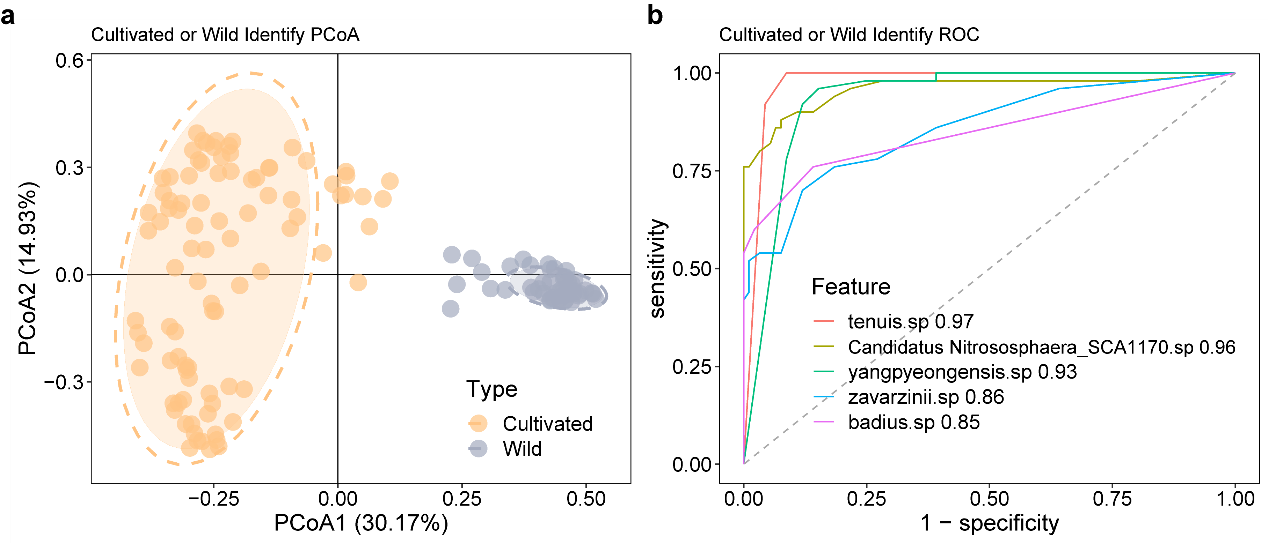


**Figure S5.** **Prediction of the plant growth condition based on root-associated microbial community composition.** (**a**) Five species including *tenuis*, candidatus *Nitrosophaera*_*SCA1170*, *yangpyeogensis,* *zavarzinii* and *badius* can distinguish the cultivation and wild samples. (**b**) Comparison of receiver operating characteristic (ROC) curves of the five species in the random forest prediction test.


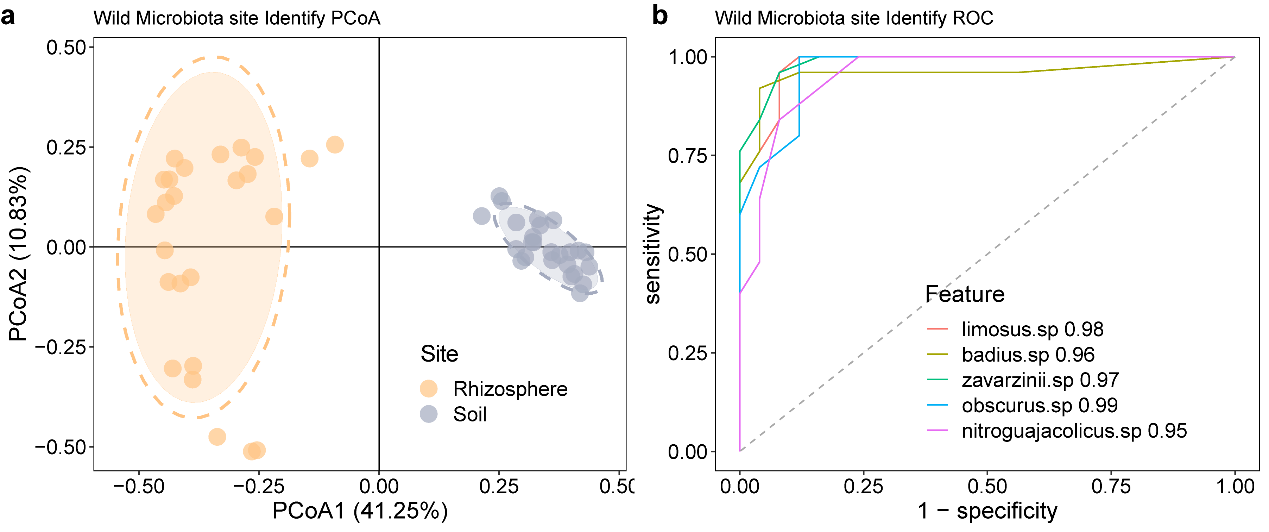


**Figure S6.** **Prediction of the origin site of wild *G. uralensis* based on the root-associated microbial community composition.** (**a**) Five species including *limosus*, *badius*, *zavarzinii*, *obscurus*, and *nitroguajaclicus* can distinguish the rhizosphere and soil samples. (**b**) Comparison of receiver operating characteristic (ROC) curves of the five species in the random forest prediction test.


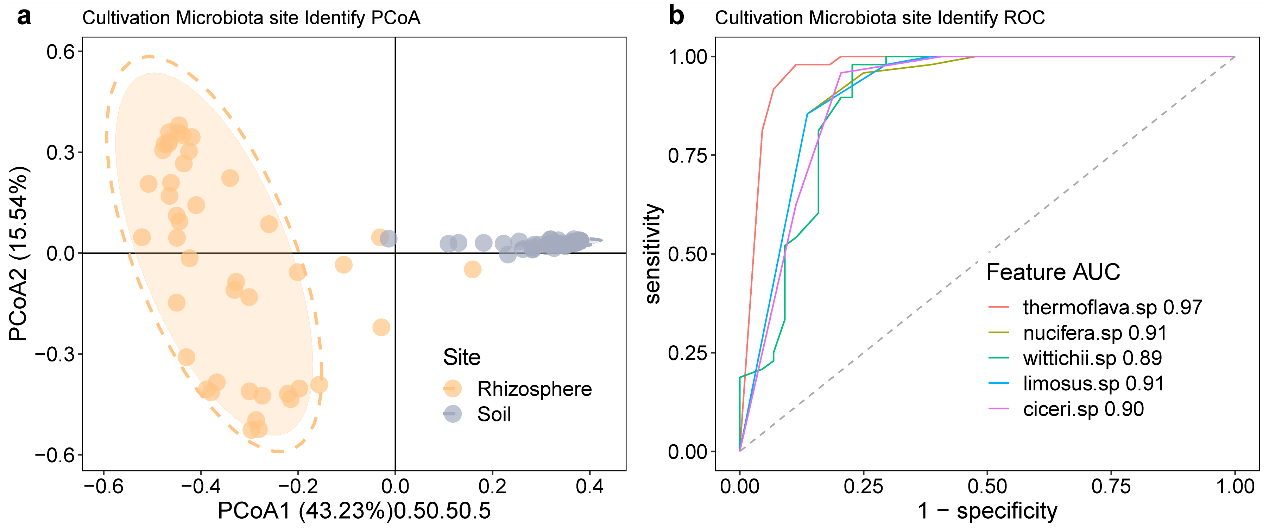


**Figure S7.** **Prediction of the origin site of cultivated *G. uralensis* based on the root-associated microbial community composition.** (**a**) Five species including *thermoflava*, *nucifera*, *wittichii*, *ciceri*, and *limosus* can distinguish the rhizosphere and soil samples. (**b**) Comparison of receiver operating characteristic (ROC) curves of the five species in the random forest prediction test.


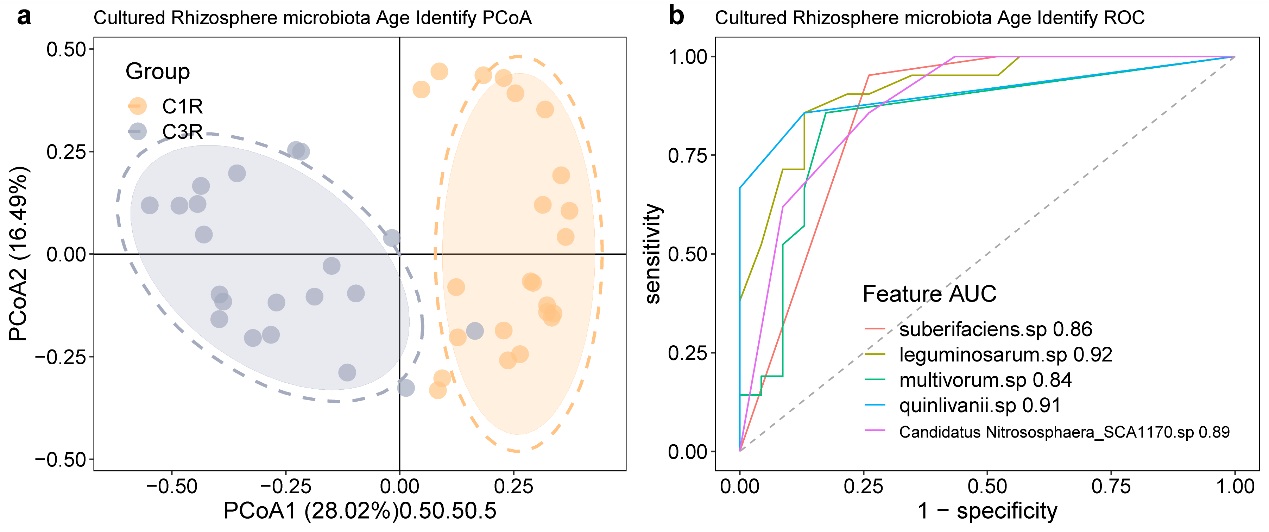


**Figure S8.** **Prediction of the growth year of cultivated *G. uralensis* based on the rhizosphere microbiota microbial community composition.** (**a**) Five species including *suberifaciens*, *leguminosarum*, *multivorum*, *quinlivanii* and Candidatus *Nitrososphaera*_SCA1170 can distinguish the different growth year samples. (**b**) Comparison of receiver operating characteristic (ROC) curves of the five species in the random forest prediction test.


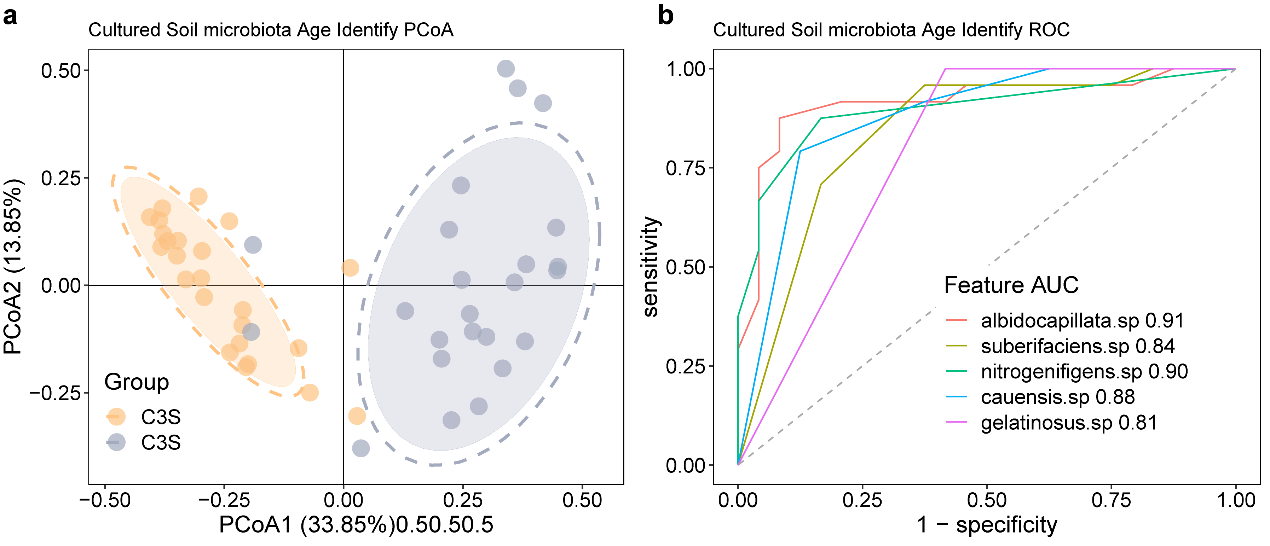


**Figure S9. Prediction of the growth year of** **cultivated *G. uralensis* based on soil microbial community composition.** (**a**) Five species including *albidocapillata*, *suberifaciens*, *nitrogenifigens*, *cauensis*, and *gelatinosus* can distinguish the different growth year samples. (**b**) Comparison of receiver operating characteristic (ROC) curves of the five species in the random forest prediction test.

# References

1. Chen, C., R. He, Z. Cheng, M. Han, Y. Zha, P. Yang, et al., *The Seasonal Dynamics and the Influence of Human Activities on Campus Outdoor Microbial Communities.* Frontiers in Microbiology, 2019. **10**: p. 1579.

2. Langille, M.G.I., J. Zaneveld, J.G. Caporaso, D. Mcdonald, K. Dan, J.A. Reyes, et al., *Predictive functional profiling of microbial communities using 16S rRNA marker gene sequences.* Nature Biotechnology, 2013. **31**(9): p. 814.

3. Breiman, L., *Random Forests.* Machine Learning, 2001.

4. Robin, X., N. Turck, A. Hainard, N. Tiberti, F. Lisacek, J.C. Sanchez, et al., *pROC: an open-source package for R and S+ to analyze and compare ROC curves.* BMC Bioinformatics, 2011. **12**(1): p. 77.
